# Changing the Landscape of Opiate Addiction Treatment - the Impact of Long Acting Injectable Buprenorphine (Buvidal) and the Buvidal Psychological Support Services (BPSS) on the Reduction of Illicit Drug Use

**DOI:** 10.1192/bjo.2026.11638

**Published:** 2026-06-30

**Authors:** Merin Jacob, Jan Melichar

**Affiliations:** 1 Cardiff University School of Medicine, Cardiff, United Kingdom; 2 Cardiff and Vale UHB Psychiatry, Cardiff, United Kingdom

## Abstract

**Aims:**

Long acting injectable buprenorphine (LAIB – Buvidal^TM^) is a well-recognised, effective licensed opioid substitution therapy (OST). It works as a partial mu-opioid receptor agonist and kappa opioid antagonist. The Buvidal Psychological Support Service (BPSS) is a rapid access organisation service, offering a 3-tiered trauma informed psychological support system for those on LAIB in Cardiff.

-To establish the impact of the BPSS for those on LAIB treatment. With the objective to analyse therapy status, retention on Buvidal opposed to alternative OST and coinciding illicit drug use other than opioids of those referred.

-To determine whether completion of BPSS Tier 1 (8 sessions) is associated with a decrease in long term opioid and overall illicit drug use.

-To determine LAIB treatment retention vs other OSTs.

**Methods:**

Use of PARIS database to obtain data on 289 BPSS referrals to date. Quantitative data on therapy status, date of initial and latest LAIB dose and current substance use was obtained and analysed. Qualitative data on the status of referrals was also obtained. Individualised chi-squared tests were conducted, significance set to p <0.05 to determine the association between the completion of Tier 1 of the BPSS service and a reduction in illicit drug use. Categorical data of each referral from the PARIS database was used to record individual illicit drug use.

**Results:**

Those completing Tier 1, showed significantly lower illicit opioid use (1.5% vs 10.5%, p <0.05 ) and overall illicit drug use (48.4% vs 20.2%, p <0.05) compared to those not engaged or discharged from the service. Alcohol, benzodiazepine, cocaine, crack, cannabis and gabapentin use did not differ between those who completed Tier 1 to those discharged/not engaged (p >0.05).

**Conclusion:**

Those on LAIB – Buvidal treatment as an opioid substitute medication can be referred to the BPSS. Of those referred retention of Buvidal as the opioid substitution treatment, is markedly higher than that of alternative OSTs. Completion of the Tier 1 BPSS service can be associated with an increased likelihood of being free of illicit drug use and reduced opiates use. Alcohol, benzodiazepine, cocaine, crack, cannabis and gabapentin use did not differ between those who completed Tier 1 compared to discharged, this perhaps associated with lack differentiation between self-medication or recreational use. Despite this, completion BPSS Tier 1 can be strongly associated with a decrease in illicit drug use, therefore long-term, more comprehensive research could be vital in reinforcing this potential.

